# A New EGFR Inhibitor from *Ficus benghalensis* Exerted Potential Anti-Inflammatory Activity via Akt/PI3K Pathway Inhibition

**DOI:** 10.3390/cimb44070205

**Published:** 2022-07-02

**Authors:** Rania Alaaeldin, Heba Ali Hassan, Islam M. Abdel-Rahman, Reham H. Mohyeldin, Nancy Youssef, Ahmed E. Allam, Sayed F. Abdelwahab, Qing-Li Zhao, Moustafa Fathy

**Affiliations:** 1 Department of Biochemistry, Faculty of Pharmacy, Deraya University, Minia 61111, Egypt; rania.alaadin@deraya.edu.eg; 2 Department of Pharmacognosy, Faculty of Pharmacy, Sohag University, Sohag 82524, Egypt; heba.ali@pharm.sohag.edu.eg; 3 Department of Pharmaceutical Chemistry, Faculty of Pharmacy, Deraya University, Minia 61111, Egypt; dr.islam_moh@deraya.edu.eg; 4 Department of Pharmacology & Toxicology, Faculty of Pharmacy, Deraya University, Minia 61111, Egypt; reham.hassan@deraya.edu.eg; 5 Department of Clinical Pathology, Faculty of Medicine, Minia University, Minia 61512, Egypt; n.youssef@worc.ac.uk; 6 Department of Pharmacognosy, Faculty of Pharmacy, Al-Azhar University, Assiut 71524, Egypt; ahmedallam@azhar.edu.eg; 7 Department of Pharmaceutics and Industrial Pharmacy, College of Pharmacy, Taif University, Taif 21944, Saudi Arabia; s.fekry@tu.edu.sa; 8 Department of Radiology, Graduate School of Medicine and Pharmaceutical Sciences, University of Toyama, Toyama 930-0194, Japan; 9 Department of Biochemistry, Faculty of Pharmacy, Minia University, Minia 61519, Egypt; 10 Department of Regenerative Medicine, Graduate School of Medicine and Pharmaceutical Sciences, University of Toyama, Toyama 930-0194, Japan

**Keywords:** anti-inflammatory activity, cyclooxygenases, iNOS, EGFR, Akt/PI3K pathway

## Abstract

Inflammation is a critical defensive mechanism mainly arising due to the production of prostaglandins via cyclooxygenase enzymes. This study aimed to examine the anti-inflammatory activity of fatty acid glucoside (FAG), which is isolated from *Ficus benghalensis* against lipopolysaccharide (LPS)-stimulated RAW 264.7 macrophages. The cytotoxic activity of the FAG on RAW 264.7 macrophages was evaluated with an MTT assay. The levels of PGE2 and NO and the activity of iNOS, COX-1, and COX-2 enzymes in LPS-stimulated RAW 264.7 cells were evaluated. The gene expression of *IL-6*, *TNF-*α, and *PGE2* was investigated by qRT-PCR. The expression of epidermal growth factor receptor (EGFR), Akt, and PI3K proteins was examined using Western blotting analysis. Furthermore, molecular docking of the new FAG against EGFR was investigated. A non-cytotoxic concentration of FAG increased NO release and iNOS activity, inhibited COX-1 and COX-2 activities, and reduced PGE2 levels in LPS-stimulated RAW 264.7 cells. It diminished the expression of *TNF-α*, *IL-6*, *PGE2*, EGFR, Akt, and PI3K. Furthermore, the molecular docking study proposed the potential direct binding of FAG with EGFR with a high affinity. This study showed that FAG is a natural EGFR inhibitor, NO-releasing, and COX-inhibiting anti-inflammatory agent via EGFR/Akt/PI3K pathway inhibition.

## 1. Introduction

Inflammation is a crucial, specific, and self-controlled defensive mechanism that is triggered by the host in response to microbial infection, tissue injury, or trauma. However, when the triggering factors are not contained, inflammation can be associated with a dysregulation of the normal physiological processes, and the escalated response can cause serious chronic inflammatory disorders [1,2,3,4]. Inflammation can contribute to the pathogenesis of arthritis, stroke, cancer, neurodegenerative, and cardiovascular diseases [5,6,7,8,9]. Prostaglandins (PGs) are key regulators in the generation of the inflammatory response. PGs are synthesized from the 20-carbon unsaturated arachidonic acid by cyclooxygenase (COX) enzymes. Prostaglandin E2 (PGE2) is the most abundant PG throughout the body, which contributes to all processes involved in the classical signs of inflammation, such as swelling, redness, and pain [10]. It mediates arterial dilatation and microvascular permeation to increase blood flow to the inflamed area, leading to redness and oedema [10].

A member of the tyrosine kinase receptor family is the epidermal growth factor receptor (EGFR), which is implicated in various physiological processes including cellular proliferation, differentiation, adhesion, migration, and apoptosis [11,12]. Upon ligand binding, an auto-phosphorylation and activation of the cytoplasmic domain of the tyrosine kinase occurs, leading to activated EGFR; phosphorylated EGFR (pEGFR). The activated EGFR stimulates many signal transduction pathways, including the protein kinase B (Akt)/phosphoinositide 3-kinases (PI3K) pathway, which is involved in various biological processes [13,14,15]. Recently, a new approach was proposed for targeting and inhibiting EGFR to attenuate inflammation [16].

Currently, most of the anti-inflammatory agents in clinical use are non-steroidal anti-inflammatory drugs (NSAIDs), which target the two isoforms of cyclooxygenases: COX-1 and COX-2 to exert anti-inflammatory actions. However, NSAIDs applications are limited by some side effects such as bronchospasm, thrombosis, gastrointestinal bleeding, and renal failure [17]. Accordingly, extensive studies are underway to identify and develop natural products that can be used as antioxidant and anti-inflammatory agents [18,19,20,21,22].

A new fatty acid glucoside (FAG), recently isolated from the ethyl acetate extract of *Ficus benghalensis* fresh leaves by Hassan et al., was shown to exhibit antioxidant and anti-Alzheimer activity [23]. In this study, we aim to investigate the anti-inflammatory activity of this new FAG against lipopolysaccharide (LPS)-stimulated RAW 264.7 murine macrophage cells on a molecular basis by inspecting the EGFR/Akt/PI3K signaling pathway.

## 2. Materials and Methods

### 2.1. FAG

**Figure 1:**
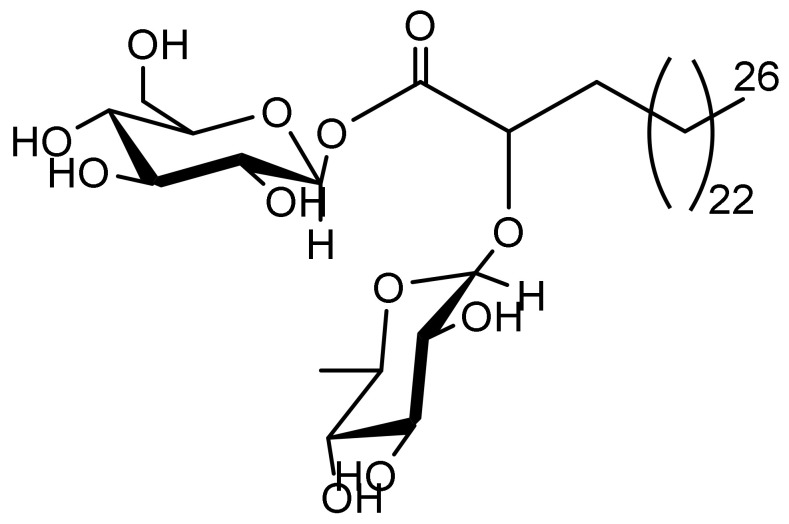
Chemical structure of the new FAG [2-O-α-L-rhamnopyranosyl-hexacosanoate-β-D-glucopyranosyl ester] isolated from the ethyl acetate extract of *Ficus benghalensis* fresh leaves.

FAG [2-*O*-*α*-L-rhamnopyranosyl-hexacosanoate-*β*-D-glucopyranosyl ester] was isolated from the ethyl acetate extract of *Ficus benghalensis* fresh leaves and identified by ^13^C-NMR and ^1^H-NMR spectral data as described [23]. The chemical structure of the new FAG is shown in Figure 1.

### 2.2. RAW 264.7 Cell Culture

The RAW 264.7 murine macrophage cells were purchased from American Type Culture Collection (ATCC, Manassas, VA, USA). The cells were cultured at 37 °C in Roswell Park Memorial Institute (RPMI)-1640 complete medium (Sigma-Aldrich, Inc., St. Louis, MO, USA), supplemented with 10% fetal bovine serum (FBS) (Sigma Aldrich Inc.) and antibiotics (100 U/mL penicillin, and 100 μg/mL streptomycin) (Sigma Aldrich Inc.) in a 5% CO_2_ humified incubator.

### 2.3. Cell Viability Assay

An MTT [3-(4, 5-dimethyl thiazol-2yl)-2, 5-diphenyltetrazolium bromide] assay was used to estimate cell viability. At a density of 1 × 10^4^, RAW 264.7, the cells were cultured in 96-well plates for 24 h. Afterward, the cells were treated with the FAG at concentrations of 0.55, 2.2, 8.7, 34.6, and 138.7 nM for two hours, then lipopolysaccharide (LPS) (Salmonella abortus equii, Sigma Aldrich, Inc.) was induced at a concentration of 1 μg/mL for 18 h. The cells were incubated for 4 h after the addition of MTT solution (5 μL/well). The supernatant was removed, then 100 μL of dimethyl sulfoxide (DMSO) was added. At 570 nm, the absorbance was estimated using an ELISA plate reader [24]. The experiments were performed in triplicate.

### 2.4. Determination of NO, iNOS, PGE2, COX-1 and COX-2

At a density of 2.5 × 10^5^, RAW264.7, the cells were cultured for 24 h in a 24-well plate in a complete RPMI-1640 medium. Then, the cells were treated for two hours with the new FAG (8.7 nM) before stimulation for 18 h with 1 μg/mL of LPS. Indomethacin was used as a positive control at a concentration of 5 μM [25]. A nitric oxide (NO) microplate assay kit (#MBS8243214, MyBioSource, Inc., San Diego, CA, USA) was used to measure NO secretion. iNOS simple step ELISA kit (ab253219, Abcam, Cambridge, MA, USA) was used to measure Mouse inducible nitric oxide synthase (iNOS) activity. A PGE2 ELISA kit (#ab133021, Abcam) was used to measure the PGE2 concentration. A COX-1 ELISA kit (#MBS753621, MyBioSource, Inc.) was used to measure COX-1 activity. A COX-2 ELISA kit (#MBS266603, MyBioSource, Inc.) was used to measure COX-2 activity. A standard curve was acquired according to the manufacturer’s instructions. For iNOS, COX-1, and COX2, the absorbance was measured at 450 nm, and for NO and PGE2, it was measured at 550 and 405 nm (correction wavelength set at 570–590), respectively, using an ELISA plate reader. From the standard curves, the concentrations of NO and PGE2 and the activities of iNOS, COX-1, and COX-2, were estimated. The experiments were performed in triplicate.

### 2.5. In Vitro Determination of COX-1 and COX-2 Enzymatic Activity

To further evaluate the anti-inflammatory activity of the new FAG, COX-1 and COX-2 enzyme activities were evaluated in vitro. A COX-1 inhibitor screening kit (#K548-100, BioVision Inc., Alexandria, Egypt) was used to measure the in vitro COX-1 enzymatic activity, while the COX-2 inhibitor screening kit (#K547-100, BioVision Inc.) was used to measure in vitro COX-2 enzymatic activity. The FAG was used at different concentrations: 0.13, 1.3, 13.8, and 138.7 nM. Indomethacin was used as a positive control at the concentrations of 0.27, 2.7, 27.9, and 279.4 nM. According to the manufacturer’s instructions, 10 μL of FAG or indomethacin was added to each well, and 80 μL of reaction master mix was prepared (76 μL of COX buffer assay, 1 μL COX probe, 2 μL diluted COX cofactor, 1 μL COX1 or COX2) and added to each well, and the fluorescence was measured at Ex/Em = 535/587 nm. The experiments were performed in triplicate. The relative percentage of inhibition of COX-1 and COX-2 was calculated according to the following Equation:

$$\%\;Relative\;inhibtion=\frac{Absorbance\;of\;EC-Absorbance\;of\;S}{Absorbance\;of\;EC}\times 100$$

where *EC* is enzyme control, and *S* is the sample.

### 2.6. Quantitative Real Time-Polymerase Chain Reaction Assay

RAW264.7 cells were treated with the new FAG (8.7 nM) for 2 h before the stimulation for 18 h with LPS (1 μg/mL). As a positive control, indomethacin (5 μM) was used. Then, total RNA extraction was performed on the cells according to the Qiagen RNA extraction kit instructions (Hilden, Germany). The expression of the *tumour necrosis factor-alpha* (*TNF-α*), *interleukin-6* (*IL-6*), and *PGE2* genes was assessed by real-time qPCR. Quantification of mRNA was achieved by utilizing the Rotor-Gene 6000 Series Software 1.7. As an internal control, *Glyceraldehyde 3-phosphate dehydrogenase* (*GAPDH)* was used [26]. Table 1 shows the sequences of the used primers, which were obtained from the National Centre for Biotechnology Information (NCBI). Using the Qiagen one-step RT-PCR (Qiagen), the RT-PCR reactions were performed. Each reaction contains total RNA (100 ng), 1x buffer, dNTP (400 μM), forward and reverse primers (0.6 μM), and the enzyme mix (2 μL). The conditions were as follows: 35 cycles of denaturation step for 25 s at 95 °C, primer annealing for 30 s at 58 °C, and polymerization step for 20 s at 72 °C. The experiment was performed in triplicate. For each sample, the analysis determined the cycle threshold (Ct) and calculated the average Ct. Using the SYBR Green fluorescent dye and the Rotor-Gene 6000 Series Software 1.7; melting curve analysis was performed using 1 °C intervals between 60 and 95 °C to avoid contamination and exclude the generation of non-specific products. The target gene expression in the treated cells was calculated after normalization to *GAPDH* expression relative to the untreated ones.

### 2.7. Western Blot Analysis

Sodium dodecyl sulphate–polyacrylamide gel electrophoresis (SDS-PAGE) analysis was performed to examine the expression of phosphorylated and total EGFR, Akt, and PI3K proteins. RAW264.7 cells were treated with the new FAG (8.7 nM) for two hours before stimulation for 18 h with LPS (1 μg/mL). At first, the samples were washed with PBS, followed by protein extraction in RIPA lysis buffer, supplemented with the protease inhibitor cocktail (Roche, Mannheim, Germany). RIPA lysis buffer contains NaCl (150 mM), Tris–Cl (50 mM), pH 7.5; SDS (0.1%), PMSF (1 mM), sodium deoxycholate (0.5%), and Nonidet P-40 (1%). To estimate the protein concentration in each sample, the Bradford method was used [27]. The proteins (30 μg) were separated using 15% acrylamide of SDS-PAGE. A Hybond™ nylon membrane (GE Healthcare) was used for sample transference, and it was incubated in Blocking Solution at room temperature for 1 h. An overnight incubation for all membranes was performed with EGFR, Akt, and PI3K antibodies (New England Biolabs, Ipswich, MA, USA) diluted (1:1000) with PBS. Membrane washing for 30 to 60 min was performed, followed by a 1 h incubation with the HRP-conjugated secondary antibody (New England Biolabs) diluted (1:1000) in PBS at room temperature [28]. A luminescent image analyzer (LAS-4000, Fujifilm Co., Tokyo, Japan) and an enhanced chemiluminescence kit (GE Healthcare, Little Chalfont, UK) were used to detect the immunoreactive proteins, according to the manufacturer’s instructions. As an internal control, β-actin antibody (New England Biolabs) diluted in PBS at 1:1000 was used. A Bio-Rad Trans-Blot SD Cell apparatus (Bio-Rad, Hercules, CA, USA), using a discontinuous buffer system, was used for electrophoresis and electroblotting. The Image Processing and Analysis Java (ImageJ) program was used for densitometric analysis. The data were normalized to β-actin levels.

### 2.8. Molecular Docking Study of the New FAG into EGFR Binding Site

The X-ray crystallographic three-dimensional structure of the protein target EGFR catalytic domain in the complex with erlotinib was downloaded as a Pdb file under code (1M17) from the database and was stored in a Protein Data Bank on the internet (http://www.rcsb.org/pdb (1 February 2022)). All of the docking studies, simulations, and algorithmic calculations were performed using the drug discovery platform of the Molecular Operating Environment 2019.0102 software (MOE). The protein was initially prepared by removing the water molecules and unincluded ligands, then by launching the quick preparation tool, which was incorporated into MOE 2019, utilizing the default options presented in it. After validating the docking method, the selected compounds, stored as MDB files, were docked on the prepared protein. The methodology of the docking procedure includes, firstly, the selection of the active site of the protein by applying the site finder tool in the operating program, MOE, followed by running the docking tool. The algorithmic parameters were adjusted by choosing the docking site as the ligand atoms and the placement methodology used as the alpha triangle, while the scoring methodology was optimized to its default values as London dG. The receptor–ligand interactions of the complexes were examined as 2D interactions and 3D conformations. The poses of the complexes that showed high affinity to the docked receptor with the best ligand–enzyme interactions were selected and stored for energy calculations. The selection of poses was conducted according to their obtained binding scores and RMSD Refine values. The obtained scores, interactions with the binding pocket site of the enzymes, and RMSD Refine values were discussed.

### 2.9. Statistical Analysis

To obtain the results, at least three independent experiments were performed. The data were expressed as mean ± standard deviation. Using GraphPad Prism 9 statistical software (GraphPad) and Excel software (Microsoft, Redwood, WA, USA), a Student’s *t*-test was used to analyze differences after a one-way analysis of variance (ANOVA). When the probability values (*p*) were < 0.05, the differences were considered significant.

## 3. Results

### 3.1. Cytotoxic Activity of the New FAG

**Figure 2:**
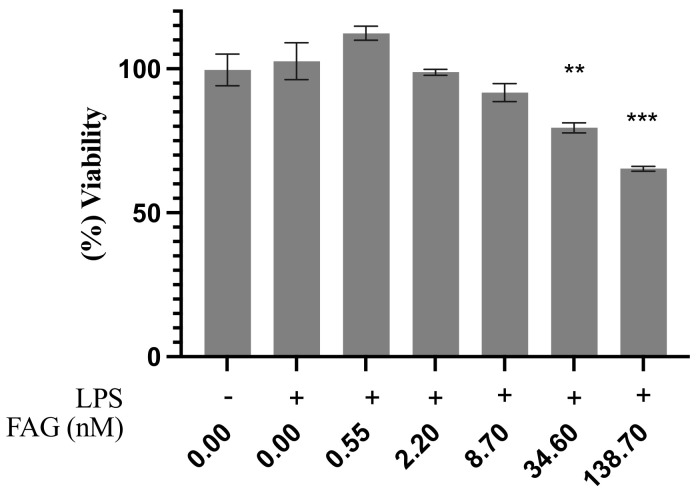
Cytotoxic evaluation of the new FAG on RAW264.7 cells. FAG was added two hours before induction with LPS. Percentage of viability was expressed relative to that of LPS-untreated RAW 264.7 cells. Bars represent mean ± SD (*n* = 3), **; *p* < 0.01, ***; *p* < 0.001, compared to untreated LPS-stimulated RAW 264.7 cells.

The cytotoxic activity of FAG was examined against LPS-stimulated RAW 264.7 cells using a cell viability assay before a further assessment of its anti-inflammatory activity on LPS-stimulated RAW 264.7 cells. Figure 2 shows that there was no notable cytotoxic activity observed at the concentrations of 0.55, 2.2, and 8.7 nM, while at the concentrations of 34.6 and 138.7 nM, the percentage of viable cells was significantly (*p* < 0.01 and *p* < 0.001, respectively) decreased to 79.5 % ± 1.7 and 65.3% ± 0.86, respectively.

### 3.2. The Effect of FAG on of NO, iNOS, PGE2, COX-1 and COX-2

**Figure 3:**
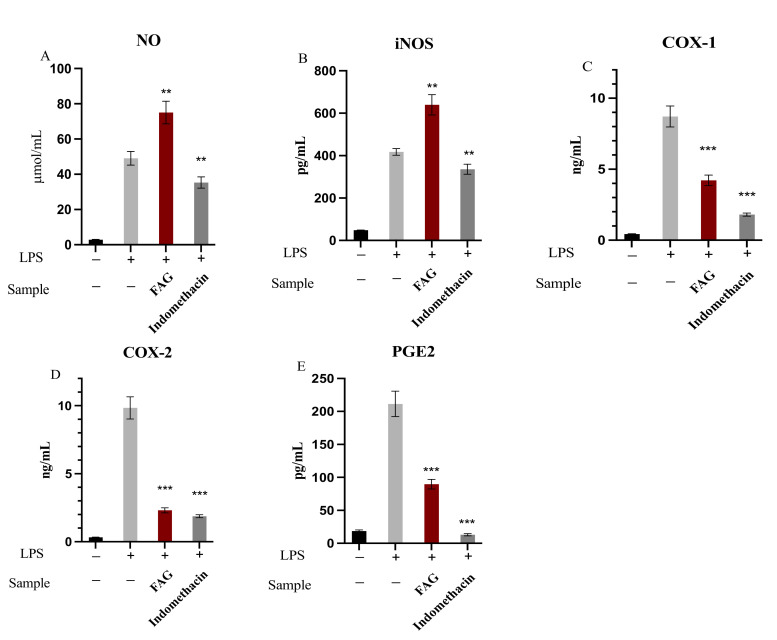
The effect of the FAG on NO concentration (**A**), iNOS enzyme activity (**B**), COX-1 enzyme activity (**C**), COX-2 enzyme activity (**D**), and PGE2 concentration (**E**) before and after treatment of LPS-stimulated RAW264.7 cells with the new FAG. Indomethacin was used as a positive control. Bars represent mean ± SD (*n* = 3), **; *p* < 0.01, ***; *p* < 0.001, when compared to untreated LPS-stimulated RAW264.7 cells.

The anti-inflammatory activity of FAG (8.7 nM) was examined through the determination of the concentrations of NO and PGE2 and an assessment of the activities of the iNOS, COX-1, and COX-2 enzymes after the treatment with LPS in RAW 264.7 cells. As shown in Figure 3, FAG significantly increased (*p* < 0.01) the release of NO and iNOS enzyme activity to 75.02 ± 6.41 μmol/mL and 639.6 ± 48.2 pg/mL, respectively, when compared to LPS-stimulated RAW 264.7 untreated cells. While the activity of COX-1 and COX-2 enzymes was significantly decreased (*p* < 0.001) after the treatment with FAG to 4.21 ± 0.37 ng/mL and 2.31 ± 0.18 ng/mL in LPS-stimulated RAW 264.7 cells, respectively, compared to LPS-stimulated RAW 264.7 untreated cells. Furthermore, the FAG significantly decreased (*p* < 0.001) the PGE2 concentration to 89.6 ± 7.3 pg/mL compared to the LPS-stimulated RAW 264.7 untreated cells.

### 3.3. In Vitro Inhibition of COX-1 and COX-2 Enzymatic Activity

**Figure 4:**
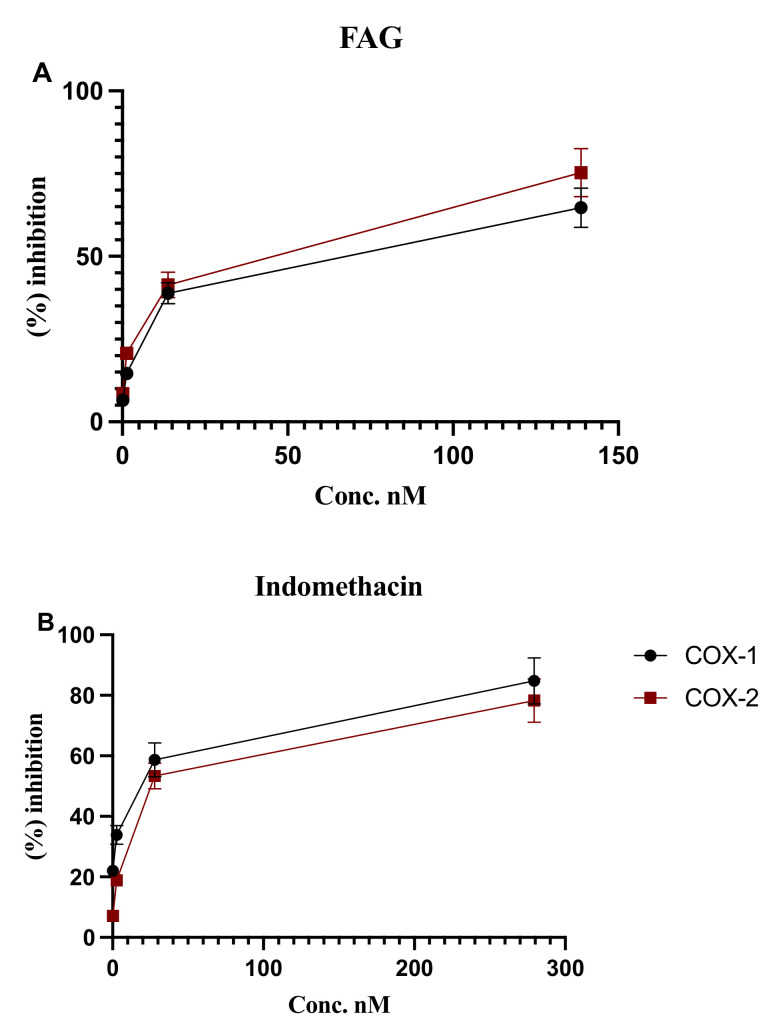
The in vitro inhibitory effect of (**A**) FAG on COX-1 and COX-2 enzymes activity at the concentrations of 0.13, 1.3, 13.8, and 138.7 nM. (**B**) Indomethacin was used as a positive control at the concentrations of 0.27, 2.7, 27.9, and 279.4 nM. Bars represent mean ± SD (*n* = 3).

To confirm the inhibitory effect of the new FAG on COX-1 and COX-2 enzyme activity, the in vitro evaluation of their activities was further examined. As shown in Figure 4, FAG increased the percentage of the inhibition of COX-1 and COX-2 enzymes in a concentration-dependent manner. At a concentration of 138.7 nM, COX-1 and COX-2 enzyme activity were inhibited by 64.7 ± 5.9% and 75.3 ± 7.3%, respectively.

### 3.4. Effect of the FAG on the Expression of TNF-α, IL-6 and PGE2 Genes in LPS-Stimulated RAW 264.7 Cells

**Figure 5:**
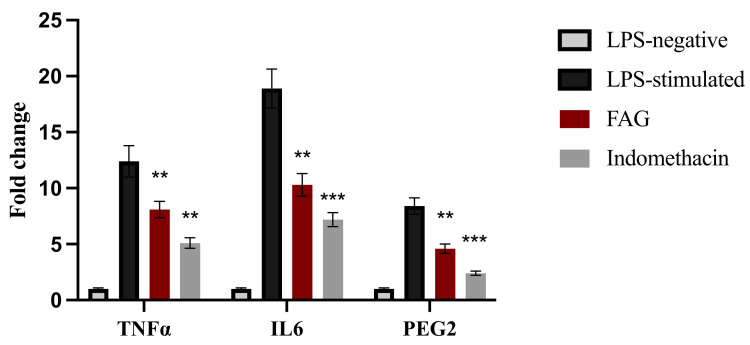
Expression of *TNF-α, IL-6*, and *PGE2* genes as determined by quantitative real-time PCR. Genes expression was normalized to the corresponding *GAPDH* gene expression and expressed relative to that of LPS-untreated RAW 264.7 cells. Indomethacin was used as a positive control. Bars represent mean ± SD. Significant difference was analyzed by one-way ANOVA. Where **; *p* < 0.01, ***; *p* < 0.001, compared to LPS-stimulated RAW 264.7 cells.

To further confirm the anti-inflammatory activity of FAG (8.7 nM), the mRNA levels of *TNF-α, IL-6*, and *PGE2* were investigated. FAG significantly (*p* < 0.01) decreased the expression of *TNFα*, *IL-6*, and *PGE2* genes in LPS-stimulated RAW 264.7 cells compared to untreated LPS-stimulated RAW 264.7 cells (Figure 5). Indomethacin was used as a positive control.

### 3.5. Effect of FAG on EGFR, Akt and PI3K Proteins Expression

**Figure 6:**
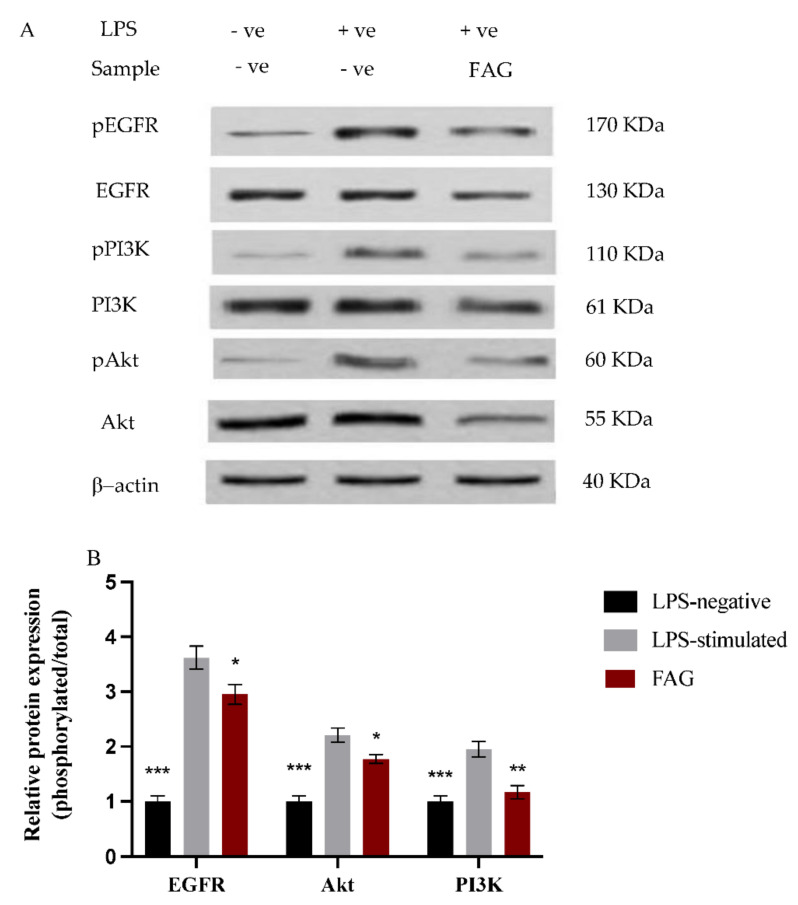
Effect of FAG on the expression of EGFR, Akt, and PI3K proteins on LPS-stimulated RAW 264.7 cells. (**A**) Representative western blots of phosphorylated and total EGFR, Akt, and PI3K proteins in LPS-stimulated RAW 264.7 cells treated with the new FAG. As an internal control, β-actin was used. (**B**) Relative phosphorylated/total protein expression ratio in RAW264.7 with/without LPS stimulation and after treatment with the FAG. Data were normalized to the corresponding β-actin protein expression and expressed relative to that of LPS-untreated RAW 264.7 cells. Bars represent mean ± SD. Significance was analyzed by one-way ANOVA test, where ***; *p* < 0.001, **; *p* < 0.01, *; *p* < 0.05, compared to untreated LPS-stimulated RAW 264.7 cells.

The phosphorylated and total protein expression of EGFR, Akt, and PI3K proteins in LPS-stimulated RAW264.7 cells was investigated by Western blotting analysis (Figure 6A). After normalization to the internal control, β-actin, the ratio of phosphorylated to total protein expression was estimated before and after the treatment with the new FAG on LPS-stimulated RAW264.7 cells (Figure 6B). The expression ratio was significantly (*p* < 0.05) reduced for EGFR and Akt proteins, while it was significantly (*p* < 0.01) decreased for PI3K protein after treatment with the LPS-stimulated RAW 264.7 cells with the FAG when compared to untreated LPS-stimulated RAW264.7 cells.

### 3.6. The New FAG Shows High Affinity towards EGFR Catalytic Domain

FAG was screened against the EGFR catalytic domain using the computational program MOE 2019.010 by performing molecular docking.

**Figure 7:**
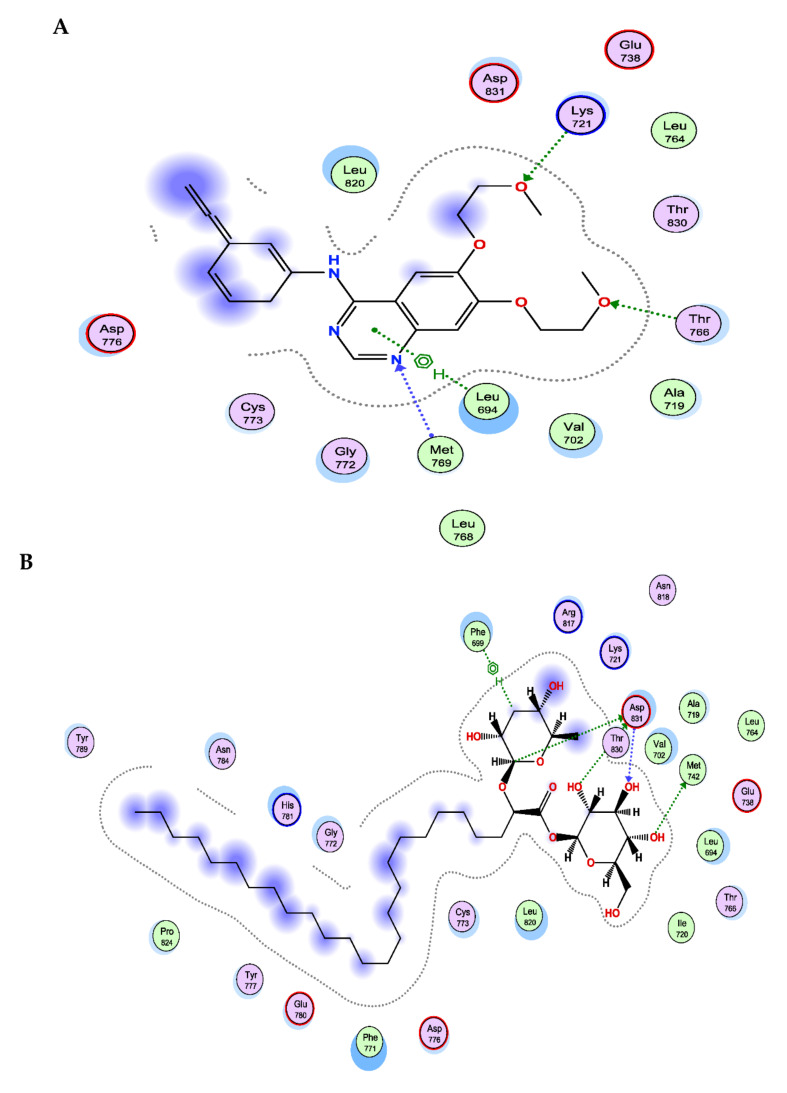

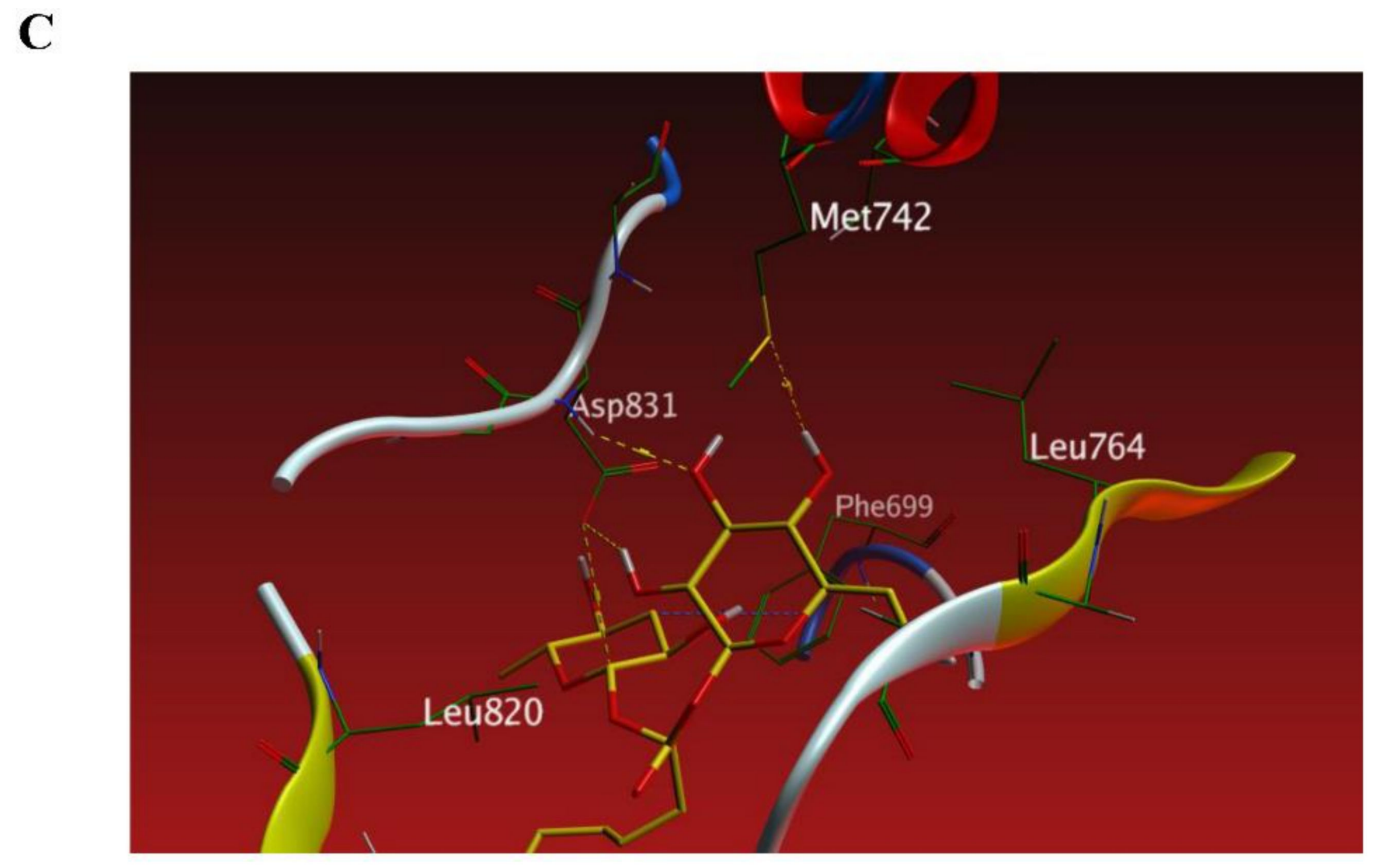
Docking results of the FAG in the active pocket site of EGFR catalytic domain, (1M17). (**A**) 2D interactions of erlotinib, (**B**) 2D interactions of FAG, and (**C**) Docking pose of FAG.

The X-ray crystallographic structure of the EGFR catalytic domain in the complex with erlotinib was obtained from the Protein Data Bank on the internet (http://www.rcsb.org/pdb/ accessed on 1 February 2022, PDB ID code 1M17). The ligand was re-docked with the active pocket site of the EGFR catalytic domain to validate our study. The docking algorithm could predict the co-crystalized ligand (erlotinib) pose with the least RMSD with an energy score of −8.074 kcal/mol showing interactions with the receptor in the form of hydrogen binding (HB) with Thr 766, Lys 721, and Met 769 residues as an H-acceptor and with amino acid Leu 694 as a hydrophobic pi-H interaction [29], as shown in Figure 7.

The docking results revealed that FAG has a high affinity toward the EGFR catalytic domain as it exhibited binding energy with a score of −11.165 kcal/mol, which is lower than the dock score of the co-crystalized ligand (erlotinib), −8.074 kcal/mol. The 2D interactions shown in Table 2 and Figure 7 displayed an HB binding mode with Met 742 and the hydroxyl group of the glucopyranosyl moiety as H-donor. Similarly, the amino acid residue, Asp831, exhibited two HB interactions, H-donor with two sugar moieties and one as an H-acceptor with the hydroxyl group of the glucopyranosyl moiety, in addition to a hydrophobic interaction with Phe 699 as an H-pi interaction.

## 4. Discussion

NSAIDs are commonly used to treat inflammation. One of the critical features of NSAIDs is their ability to release NO. NO-NSAIDs are a new class of anti-inflammatory agents with the potential to provide greater safety since they can reduce the gastrointestinal toxicity produced by classical NSAIDs [30]. NO is synthesized from L-arginine by the activity of the NOS enzyme and exhibits various physiological and pathological effects [31,32]. In particular, it contributes to the tonus and motility of the gastrointestinal system [33]. Several studies contributed to developing NO-donating and COX-inhibiting NSAIDs to provide potent anti-inflammatory activity with a protective capacity toward the gastrointestinal system [34,35,36]. The present study, for the first time, showed that this new FAG exhibited elevated NO release and iNOS enzyme activity in addition to the inhibition of COX-1 and COX-2 enzyme activity and a reduction in PGE2 levels after the treatment of LPS-stimulated RAW 264.7 cells. These findings propose and categorize this new FAG glucoside as an NO-donating and-COX inhibiting anti-inflammatory agent.

To further explain the mechanism of action of this new FAG, we investigated the expression of the EGFR protein before and after treatment on LPS- stimulated RAW 264.7 cells. EGFR has been studied extensively for developing anticancer agents [22,37,38,39,40]. Recently, several studies indicated that EGFR has a pathological role in non-neoplastic diseases. In this regard, a recent study provided strong evidence that EGFR promoted retinal vascular abnormalities, retinal dysfunction, and retinal structural impairment caused by diabetic retinopathy in mice, and the use of an EGFR inhibitor suppresses inflammation and possesses potential therapeutic targets for treating diabetic retinopathy [41]. Another recent study investigated the anti-inflammatory activity of a novel compound against LPS-stimulated RAW 264.7 cells through the inhibition of EGFR and revealed that it inhibits several inflammatory mediators, including IL-1β, IL-6, and TNF-α through the inhibition of EGFR [42]. Furthermore, an in vivo inhibition of EGFR in spinal cord injury decreased inflammation and secondary damages in a neuro-inflammation model [43]. Another study by Li et al., a cardiovascular disease model, reported that knocking down EGFR leads to an anti-inflammatory activity in cardiac muscle cells [44]. In addition, the activation of EGFR was found to contribute to inflammatory status by respiratory syncytial virus and by non-typeable *Haemophilus influenzae* [45,46]. More importantly, a study by Lin et al. linked the ATP-transactivation of EGFR to increased levels of COX-2 and phospholipase 2 enzyme activity, but high COX-2 enzyme activity was significantly reduced after treatment with an EGFR inhibitor [47].

The activation of the Akt/PI3K pathway is a downstream effector of activated EGFR. The activated Akt/PI3K pathway plays a critical role in the pathogenesis of inflammatory response, and the mechanism by which Akt/PI3K regulates inflammation is not quite understood. One recent study demonstrated that the Akt/PI3K pathway is implicated in the inflammation severity of acute pancreatitis in rats, and the mRNA levels of *Akt* and *PI3K* escalated after treatment with Akt/PI3K agonist in LPS-stimulated RAW 264.7 cells [48]. Moreover, Lin et al. investigated whether the activated Akt/PI3K pathway is implicated in high COX-2 enzyme levels through the pretreatment with Akt/PI3K inhibitor; their findings indicated that the inhibition of the Akt/PI3K pathway inhibited COX-2 enzyme activity [47]. Another study indicated that surgery-stimulated inflammatory, neuropathic pain is inhibited through the inhibition of the IL-6, TNFα, and Akt/PI3K pathways [49].

In the present study, the EGFR/Akt/PI3K pathway was activated in LPS-stimulated RAW 264.7 cells; where the ratio of phosphorylated/total protein was increased, which confirms that the initiation of inflammation is associated with higher levels of activated EGFR and a further subsequent activation of the Akt/PI3K pathway. Additionally, the mRNA levels of *IL-6* and *TNF-α*, pro-inflammatory mediators produced mainly in inflammatory models [50,51,52,53], were substantially elevated in LPS-stimulated RAW 264.7 cells. However, the EGFR/Akt/PI3K pathway was inhibited after the treatment with the new FAG; where the ratio of phosphorylated/total proteins was reduced in LPS-stimulated RAW 264.7 cells. In addition, our real-time PCR findings revealed that the FAG inhibited the expression of the *TNF-α* and *IL-6* genes in LPS-stimulated RAW 264.7 cells. A previous study reported the link between the gene deletion of EGFR and the reduction in IL-6 and TNFα in myeloid cells, which attenuates atherosclerosis [54]. This further confirms the anti-inflammatory activity exerted through the inhibition of EGFR.

**Figure 8:**
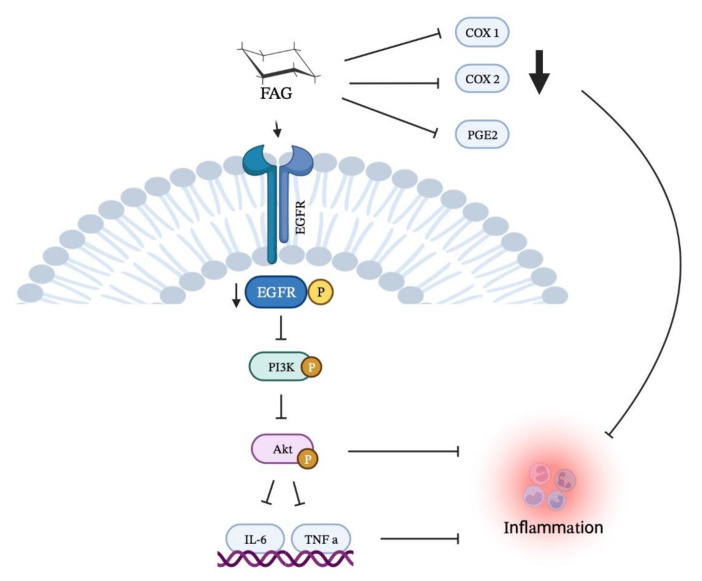
Illustrative figure summarizes the anti-inflammatory activity of FAG via the molecular inhibition of EGFR/Akt/PI3K signaling pathway.

The molecular docking analysis revealed the potential direct binding of the new FAG towards EGFR with a high affinity. This suggests that the new FAG is an EGFR inhibitor, and its anti-inflammatory action could be through the direct binding toward EGFR, resulting in decreasing the activated form of EGFR and further inhibition of the downstream signaling pathway, the Akt/PI3K pathway. Moreover, inflammatory mediators, such as IL-6 and TNF-α, were found to be suppressed. Furthermore, COX-1, COX-2, and PGE2 were downregulated after treatment with the new FAG. Looking for new therapeutic applications for existing or recently discovered candidates has received considerable attention [55,56,57,58,59,60,61]. For the first time, the present study revealed an EGFR inhibitor, NO-donating, and COX-inhibiting anti-inflammatory agent, our natural FAG, isolated from *Ficus benghalensis* leaves. The non-toxic concentration of the FAG against RAW 264.7 murine macrophage cells exerted promising molecular modulation of the EGFR/Akt/PI3K signaling pathway, as summarized in Figure 8, which prompted us to estimate the anti-cancer activity of this new compound in the future. Further investigations on human macrophages and further in vivo studies are required to elucidate the activity of this new FAG and to better understand its mechanism of action in humans.

## 5. Conclusions

The new FAG [2-*O*-*α*-L-rhamnopyranosyl-hexacosanoate-*β*-D-glucopyranosyl ester] was recently isolated from the ethyl acetate extract of *Ficus benghalensis* fresh leaves and showed promising antioxidant and anti-Alzheimer activity. This study, for the first time, examined its anti-inflammatory activity against LPS-stimulated RAW 264.7 cells. The new FAG inhibited COX-1 and COX-2 enzyme activity, reduced PGE2 levels, and increased NO release and iNOS enzyme activity. These findings categorize this new FAG within the NO-donating COX inhibitors as anti-inflammatory agents. Furthermore, this new FAG exerted an inhibitory activity on the EGFR/Akt/PI3K pathway, which has been shown to attenuate inflammation. Furthermore, the molecular docking study proposed the potential direct binding of the new FAG with EGFR with a high affinity, suggesting that it is an EGFR inhibitor that further inhibited the downstream signaling cascade of Akt/PI3K to reduce inflammation.

## Figures and Tables

**Table 1:** Sequences of the primers used.

Primer	Sequence
*TNFα*	Forward: 5′-TGTAGCCCACGTCGTAGCAAA-3′ Reverse: 5′-TGTGGGTGAGGAGCACGTAG-3′.
*IL-6*	Forward: 5′-ACCCCAATTTCCAATGCTCTCCT-3′ Reverse: 5′-GGATGGTCTTGGTCCTTAGCCAC-3′.
*PGE2*	Forward: 5′-TGTACCGAACACCCGCTGAG-3′ Reverse: 5′-GCTTTTGAGGCGCTTGCTGA-3′.
*GAPDH*	Forward: 5′-CCCAGAAGACAGTGGACGGG-3′ Reverse: 5′-CGACAGACACATCCGGGGTT-3′.

**Table 2:** Receptor interactions and binding energies of the new FAG and erlotinib into the active pocket site of EGFR catalytic domain.

NO	Compound	S ^a^ kcal/mol	RMSD_Refine ^b^	Amino Acid/Bond	Distance Å	E (kcal/mol)
1	The FAG	−11.1658	1.92	Met 742/H-donor	3.72	−1.0
				Asp 831 H-donor	3.33	−0.9
				Asp 831/H-donor	2.93	−0.5
				Asp 831/H-acceptor	3.17	−1.0
				Phe 699/H-pi	4.57	−0.6
2	Erlotinib	−8.0742	1.55	Lys 721/H-acceptor	3.06	−4.6
				Thr 766/H-acceptor	2.88	−1.1
				Met 769/H-acceptor	3.57	−0.5
				Leu 694/pi H	3.85	−0.6

^a^ S: the score of a compound placement inside the protein binding pocket. ^b^ RMSD_Refine: the root-mean-squared-deviation (RMSD) between the predicted pose and those of the crystal one (after and before refinement process, respectively).

## Data Availability

All data are fully available and included in the manuscript.
